# Infant Feeding

**Published:** 1889-11

**Authors:** 


### INFANT FEEDING.

A physician of a New York dispensary has recently made a careful study of the value of different foods customarily given to infants deprived of nursing mothers, with the following conclusions;

(1) Infants deprived of human breast milk should be fed, first of all, with cow's milk, diluted. (2) Infants artificially fed should not be fed every two hours, for the reason that more than that time, as a rule, is necessary to digest the food given. Herein is furnished the best of evidence that infants, even in the earliest days of life, ought not to be fed oftener than once in three hours.

